# Market Model for Resource Allocation in Emerging Sensor Networks with Reinforcement Learning

**DOI:** 10.3390/s16122021

**Published:** 2016-11-29

**Authors:** Yue Zhang, Bin Song, Ying Zhang, Xiaojiang Du, Mohsen Guizani

**Affiliations:** 1 The State Key Laboratory of Integrated Services Networks, Xidian University, Xi’an 710071, China; y.zhang@stu.xidian.edu.cn (Y.Z.); yzxdedm@163.com (Y.Z.); 2 Department of Computer and Information Sciences, Temple University, Philadelphia, PA 19122, USA; dxj@ieee.org; 3 Department of Electrical and Computer Engineering, University of Idaho, Moscow, ID 83844, USA; mguizani@ieee.org

**Keywords:** agent-based modelling, emerging sensor networks, Internet of Things, market model, reinforcement learning, resource allocation, topology management

## Abstract

Emerging sensor networks (ESNs) are an inevitable trend with the development of the Internet of Things (IoT), and intend to connect almost every intelligent device. Therefore, it is critical to study resource allocation in such an environment, due to the concern of efficiency, especially when resources are limited. By viewing ESNs as multi-agent environments, we model them with an agent-based modelling (ABM) method and deal with resource allocation problems with market models, after describing users’ patterns. Reinforcement learning methods are introduced to estimate users’ patterns and verify the outcomes in our market models. Experimental results show the efficiency of our methods, which are also capable of guiding topology management.

## 1. Introduction

Sensors are one of the main elements of big data ecosystems, and are critical at the data collection stage [1]. The idea of connecting sensors as sensor networks is popular and applicable in a number of applications [2]. The growing popularity of the Internet of Things (IoT) enables the communication and interaction of a huge number of devices, which also emphasizes the significance of sensors. Meanwhile, other specific networks—such as the Internet of Vehicles (IoV) [3]—are also growing rapidly, with extraordinary applications such as health care [4] and multimedia [5]. Wearable devices and other personal sensors (including smart vehicles) record our daily data, making the environment of the Social Internet of Things (SIoT) [6] and Social Internet of Vehicles (SIoV) [7] when online social networks (OSNs) are combined. With the development of smart devices, the emergence of home automation or even smart cities is not an illusion [8]. Other related fields have also contributed to the emergence of such networks. One straightforward area is wireless communication networks and device-to-device (D2D) communications [9], which can be appended to IoT [10]. This provides more opportunities for communication, accelerating the progress of a cyber-physical world with social activities, such as social-aware D2D communications [11]. Therefore, the networks raise the requirement of computing and communication technologies and systems, which are considered as emerging sensor networks (ESNs). Such networks should integrate technologies and methods with respect to resource allocation, data mining, knowledge sensing, and intelligent control in order to satisfy the variety of applications in industry and business.

An avoidable problem in ESNs is resource allocation, which intends to maximize the efficiency of whole systems [12]. This problem is more severe when conflict among agents occurs. This is the main reason why game theory is commonly considered, since the expertise of game theory is in decision-making problems with conflict, such as the prisoner’s dilemma [13]. On the other hand, not all individuals are facing conflict. For instance, somebody who enjoys meat has little likelihood of competing with a vegetarian, even though one of the resources is limited. The existence of conflict may depend on user patterns and other variables such as the quantity of data. Therefore, we take categories of data into consideration and turn to the research of users’ patterns, which is an intrinsic characteristic of people.

Users’ patterns or preferences seem too fuzzy to quantify. We merely present a naive attempt to demonstrate the function of users’ patterns without too many details. We regard users’ patterns as a set of probability, and people act according to their preference. We assume people are greedy and rational, as in game theory or other decision making methods. This means that the only concern of people is to maximize their payoffs. Thus, if we regard agents in ESNs as rational people, whose behavior is according to their preference, then the whole ESNs can be regarded as a market, since the key concept of a market is people and their activities [14]. Therefore, it motives us to borrow economic knowledge to handle resource allocation problems, because the market is famous for this capability [15].

One of the basic market models is auction models, which apply a price scheme to allocate resources. For a particular market, high production means low prices. If a firm contains some market power, such as a monopolistic firm, it is able to control the price to some degree. If the price is higher than the willingness-to-pay (typically because of the limitation of the resources), customers will leave the market. Thus, resources are allocated to the highest bidders, whose willingness-to-pay is the highest.

The market model seems complicated, due to the number of users and their interactions. Thus, we apply an agent-based modelling (ABM) method, because the rules for people are relatively straightforward, which is that people are rational and greedy. ABM is popular when a multi-agent environment is considered, such as a biological system, which focuses on the behaviors of agents and their interactions with others or with environments [16]. We consider devices as agents in biological systems, such as swarms, to study the swarm intelligence of units in our artificial environments [17]. We apply reinforcement learning methods to design the rules for agents, since they are more flexible to apply than game theory, which can only provide an analytical solution and involves the interaction of humans. Reinforcement learning is a branch of machine learning whose main concern is with decision making, which satisfies our previous discussion [18]. After a series of trial-and-error, agents can learn the best action sequences with the assistance of certain rewards. Meanwhile, due to its extraordinary performance against uncertainty, we also apply reinforcement learning methods to estimate users’ patterns, and further to solve resource allocation problems in our market model. When massive data are created by a large number of sensors, methods of data analysis under the big data environment are also required. One promising method is deep learning [19]. The key purpose is to abstract the most useful data and to eliminate redundancy. The concept of being useful, however, is fuzzy. Thus, a customization system is required, which has been discussion previously. The combination—deep reinforcement learning—has demonstrated great capability in artificial intelligence [20,21], which can be further applied to build intelligent ESNs. However, deep learning is not omnipotent, since it sacrifices computational cost to accuracy. Therefore, we focus on reinforcement learning methods, knowing that deep reinforcement learning is also a choice.

At last, security and trust management is always an unavoidable concern in the process of data mining and IoT [22]. One promising solution is to apply privacy-preserving data mining to reduce the risk at each stage of the big data ecosystem [23]. Trust management also stands for one dimension in the process of decision making, with the intuition that the resource is more reliable if the source is trustworthy. This, however, is beyond the scope of this paper. We only focus on the decision making process based on data.

Our main contribution is that we treat ESNs as markets so that we can apply ABM and market models to establish a flexible and adaptive resource allocation solution based on users’ patterns, which we have quantified and estimated. Then, we apply reinforcement learning methods to estimate users’ patterns and to solve the resource allocation problems. The key advantages of the application of reinforcement learning is its flexibility, and that it requires less participation and knowledge than that from game theory, as a comparison. The next section will introduce a market model for resource allocation. Section 3 discusses the main methods for the market model and resource allocation problem, with experimental results being presented in Section 4. Section 5 concludes the paper.

## 2. Market Model for Resource Allocation

With the growing trend of ESNs and IoT, an increasing number of devices are able to connect automatically. On the other hand, more mobile devices (such as smart phones or watches) are becoming inseparable components of our daily lives. People are becoming reliant on or rather addicted to these electronic objects. Meanwhile, social activities tend to execute with the assistance of these devices. It seems that we have avatars in a virtual world, and they may reflect our real activities and other aspects. Thus, our social patterns or other phenomena can be studied by researching the nodes and environments created by intelligent devices. Naturally, the activities of humans, being a market, can be reflected in markets in the virtual world, and this can in turn benefit our daily lives.

**Figure 1:**
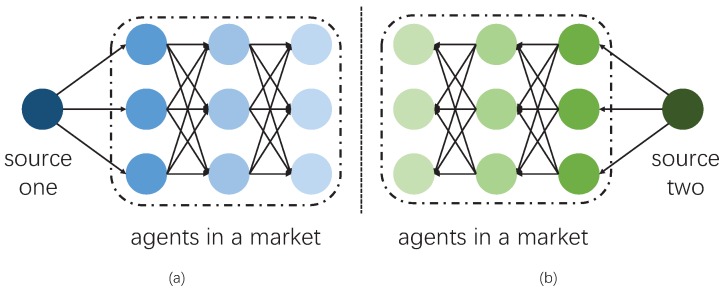
A demonstration of data flow with a hierarchical structure. Identical agents in a market are adaptable and sensitive to different categories of data, as in panels (**a**) and (**b**).

Either way, resource allocation is a popular topic in a variety of fields, such as communications and economics. The key desire is to maximize the efficiency of resources and to guarantee the quality, especially when they are rare or limited. Therefore, an order indicating the priorities of users may be obtained, according to the willingness-to-pay of all users in a particular market. This order is able to present the relationships between quantity and price, if it can be described accurately. Furthermore, topology management or data flow can be achieved with the assistance of the guidance of priorities. The most distinguishing advantage of treating conventional ESNs as markets is its flexibility. In other words, since we focus on different categories of data, the topology of ESNs should be sensitive to different data. Thus, the adaptability is required, rather than a fixed topology. Therefore, instead of building a topology scheme, we provide clues for the establishment of an efficient topology structure. One straightforward consideration is data flow. Figure 1 demonstrates two examples for such an application. According to the preferences of users, the priority is arranged towards a particular class of data. Therefore, a naturally hierarchical structure is obtained. Agents in the same layer are competitors, and all of them are potential providers for those in the next layer. This idea is similar to artificial neural networks. Due to the complication of the environment, the links among nodes are not fixed. When data belonging to a different class occur, the structure and priority are adjusted dynamically. Notice that we are not designing methods for topology management. We aim at resource allocation problems, and topology is the natural outcome of our methods. Our methods are capable of a dynamic situation. Therefore, the design of fixed topology management methods is not required.

One straightforward assumption in our model is that people are rational, since we are facing decision making problems. This assumption—which is popular among microeconomic theory and game theory—states that people will make decisions according to their preferences instead of choosing randomly. Meanwhile, prices in a market depend on a number of aspects. Our naive model only sticks to quantity, which is the main variable. This assumption may be sufficient to reveal the main relationships in markets, without too many details. Notice that a number of methods from game theory may require a further assumption that knowledge is common. Our method, however, is independent of such an assumption, which improves its flexibility.

Thus, the first priority is to analyze users’ patterns, which can further decide willingness-to-pay and divide the whole market into subsets. Then, market models and price schemes can be established for the purpose of resource allocation and topology management.

### 2.1. Users’ Patterns

For a data provider, it is unreasonable and impossible to transmit data to all users, due to the concerns of necessity and efficiency. From the perspective of a user, it is also difficult to accept all data, most of which may be irrelevant. Therefore, the destination of certain data should be treated carefully. Decision making is the key problem under such circumstances. Efficient deliveries may maximize the benefits of both terminals. Therefore, we turn to consider patterns of users, which may further decide the willingness-to-pay and guide the transmission of data.

Users’ patterns are popular when customization is taken into consideration, where the satisfaction of each individual is involved separately, instead of being treated indiscriminately. The decisional factor for patterns remain mysterious; meanwhile, the mapping from genes to patterns seems too fuzzy to be reliable. Therefore, we define users’ patterns based on users’ behaviors. Under the IoT environment, devices are more accessible to personal behavior, such as apps’ usage and habits on the internet. Thus, we may obtain a detailed description of users, which indicates their preferences.

Consider a set of data of total *N* categories, each of which could be games, business, or travelling, for instance. Notice that *N* should not be too large or too small, or the efficiency of the taxonomy is not satisfied. Thus, we quantify users’ patterns as a discrete distribution over the *N* categories. For a particular user *m*, his pattern is

(1) $$P\left(m\right)=\left\{p_{m,1},p_{m,2}...p_{m,N}\right\},\mathrm{with}\sum_{n}p_{m,n}=1$$

Under the environment of ESNs or IoT, users’ patterns play a significant role. One straightforward function is that they can define a correlation of agents, as

(2) $$\rho_{X,Y}=corr\left(X,Y\right)=corr\left(P\left(X\right),P\left(Y\right)\right)$$

**Figure 2:**
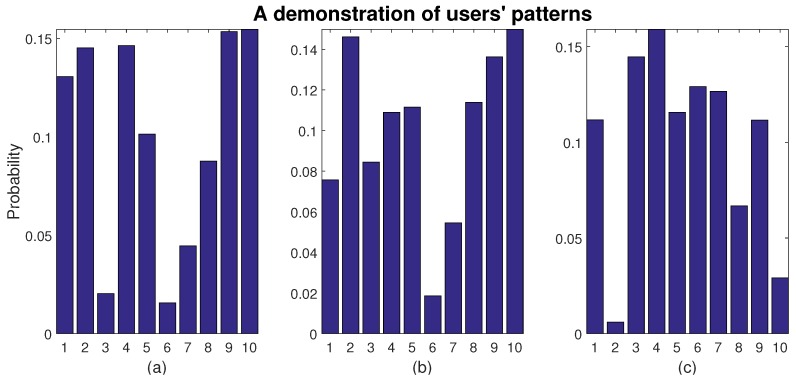
A demonstration of users’ patterns and correlations. The x-axis is the number of categories of data, whereas the y-axis is the probability. corr(P(a),P(b))=0.8064 and corr(P(a),P(c))=−0.4587, indicating the similarity of users (**a**) and (**b**) and the dissimilarity of users (**a**) and (**c**), which can also be found clearly from the figures.

A higher correlation indicates higher similarities. This can further decide the roles of agents. For instance, if agents have little correlation, they may have different preferences. Thus, when mutual data are presented, they can cooperate, since their options for maximizing payoffs do not conflict. Those who share higher correlation are more probable to compete, since they have similar desire—especially when the resource is rare. Therefore, the roles of competitors and cooperators are decided by users’ patterns, and the different roles may lead to different models, and further, to different solutions. Three examples of users’ patterns and their correlation are presented in Figure 2. Vividly, we can find that the pattern in panel (a) shares high similarity with that in panel (b), whereas panel (c) is distinguishable.

Even though users’ patterns are useful and critical, estimating users’ patterns is difficult. Its complication is mainly due to its fuzziness and invisibility. It is challenging to describe the preferences accurately, even for users themselves. Therefore, we introduce reinforcement learning—whose expertise is dealing with uncertainty—to handle this problem, the details of which are presented in Section 3.

### 2.2. Market Model and Price Scheme

Users’ patterns decide their willingness-to-pay, which is related to their economic activities. The logic is straightforward and obvious. People are more willing to pay for their preferences. Thus, one intuitive conclusion is that if the price of a product is less than the willingness-to-pay of a buyer, she is more likely to purchase it, since her satisfaction is positive. Mathematically, the reward or satisfaction of a user *m* is

(3) $$R_{m,n}=P_{n}-W_{m,n}$$

where $P_{n}$ is the price at market *n* and $W_{m,n}$ is her willingness-to-pay. Higher $R_{m,n}$ indicates more satisfaction for the particular user.

**Figure 3:**
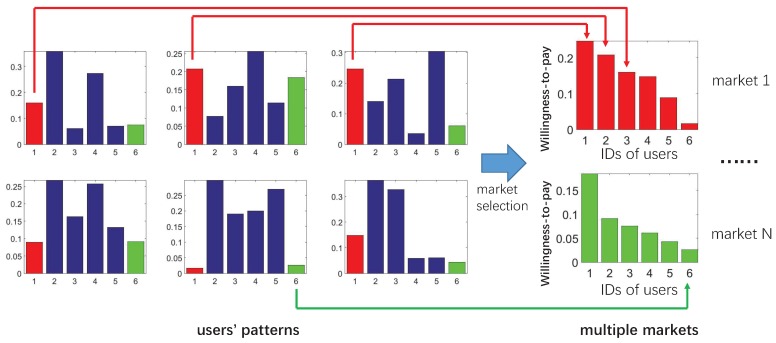
The emergence of markets based on users’ patterns. Preferences in one specific category from each user establish one market, indicated by colors.

By analyzing users’ patterns and extracting their preference, we can establish markets for each category of data, as in Figure 3. Notice that only two markets are presented as an instance. Thus, the preference of one specific user decides her role in this market. Higher willingness-to-pay means she accepts a relatively higher price for this certain category of data. We also rank the willingness-to-pay within one market in a descending order, which may reflect their priorities in this market. We divide markets subject to the categories of data, due to the fact that data are not evenly distributed over all categories. This is similar to the different numbers of providers in industry. Therefore, regarding all kinds of data as a whole is vague, thus unsuitable for the market.

After managing to build the model for markets, we turn to the discussion of price scheme, which is the key method of resource allocation in economics. One straightforward and popular price scheme is the auction model, which focuses on the relationships between the numbers of providers and consumers. Specifically, if the number of data providers is greater than demanders—meaning that data are sufficient for the market—then every customer is able to access the data. Then, the policy of providers is to lower the price. The reason is two-fold. On one hand, reducing the price and selling greater quantities until its marginal cost exceeds marginal profit is the most efficient way to maximize one’s payoffs at free markets. On the other hand, if the price of one provider exceeds that of their competitors, people have the incentive to buy from others, which means a high price may expel customers. Therefore, all the providers will price at the minimum level, which is decided by economic rules and some particular laws from governments.

**Figure 4:**
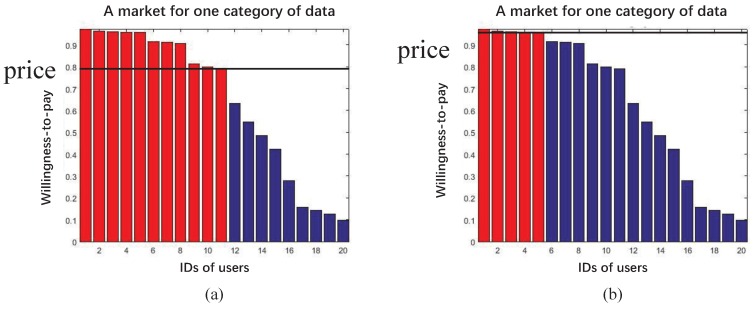
Demonstration of auction models. Prices are indicated by the horizontal dark lines. Those whose willingness-to-pay is above the price will stay (colored red), otherwise they will leave the market colored blue). Thus, when the price is higher, comparing the price in panel (b) to (a), fewer people will remain in the market.

The auction model is more interested in the situation where the capability of providing dissatisfies the ability of purchasing. In other words, goods are relatively rare. Under this scenario, the price will be higher, basically for two reasons. On one hand, reducing the price will not increase the quantities being purchased, due to the limited resource. On the other hand, the key reason why the deficiency situation occurs is that the cost of producing the product is high. Thus, the destination of the rare resource should be concerned. At an auction, the price of one particular subject keeps increasing by bidding from buyers, and finally it belongs to the highest bidder. Therefore, the auction model suggests that the price should increase until the number of data equals the number of consumers. Those whose willingness-to-pay does not fit the price will leave the market. Mathematically, let *D* denote the number of data and *C* denote the number of consumers. We are assuming that each individual purchases one piece of data at most, and the willingness-to-pay of all users is sorted in descending order. Then, when D<C, agents in the market *n* is $M_{n}=\left\{m_{1,n},m_{2,n},...,m_{D,n}\right\}$ and other members $M_{n}^{-}=\left\{m_{D+1,n},m_{D+2,n},...,m_{C,n}\right\}$ will leave the market. The price will be decided by $m_{D,n}$ and $m_{D+1,n}$. Roughly,

(4) $$W_{D+1,n}\leq P_{n}\leq W_{D,n}$$

is a valid region, since the price should satisfy $m_{D,n}$ rather than $m_{D+1,n}$, where $W_{D+1,n}$ and $W_{D,n}$ are willingness-to-pay for user D+1 and *D*, respectively. Notice that for the purpose that the price is comparable with willingness-to-pay, we normalize price into $P_{n}\in \left[0,1\right]$. Meanwhile, price within the region in Equation (4) does not affect the quantity of selling. Therefore, $P_{n}=W_{D,n}$ is the final choice for optimal profits. Figure 4 is a comparative demonstration of the auction model. Panel (b) contains less data, and therefore the higher price and fewer members in the market. The price is determined by the last member of the amended market. Even though quantities are not the sole factor affecting prices, they are still reliable references, according to economic theory. By altering prices, the limited resources are allocated to those who treasure the most, which is the natural force in economics for resource allocation tasks. Notice that—similar to the complication of users’ patterns—the willingness-to-pay of users in a market is also inaccessible. Therefore, we cannot calculate the price directly.

## 3. Agent-Based Modelling and Reinforcement Learning

Based on previous discussion, we expect to introduce a market scheme in ESNs to accomplish optimal resource allocation by economic theory. However, it is not so straightforward to apply ESNs with an interdisciplinary theory. Thus, we focus on the methods in economics, whose research targets are humans or firms. One common principle for both human networks and ESNs is that people (or devices) respond to incentives. The motivation is that each individual intends to maximize its payoffs and minimize the cost. This is the interaction of these fields. Therefore, we expect to model the environment of ESNs or IoT as a market with multiple agents, where a resource is able to be allocated according to economic theory.

### 3.1. Multi-Agent Environments and Agent-Based Modelling

One distinguishing feature of ESNs or IoT is that it connects a variety of nodes or devices. If every node is connected to the rest, the full-connect network is redundant and inefficient, since not all links are required. Meanwhile, some links may exist in an uneconomic way. Therefore, the topology management technique is required to maximize the efficiency of the networks. From the perspective of nodes or agents, this is a problem of decision making, which involves a variety of factors. Thus, we regard this environment as a multi-agent situation, and we analyze it considering decision making problems.

Typically, two standard directions to study multi-agent environments are discussed. One way is from a macro perspective, which is common in a variety of studies. Researchers and designers plan to build a comprehensive model so that every node or agent shall obey. For example, traffic laws are designed to improve traffic efficiency and to reduce accidents, if everyone obeys. Macro methods are relatively straightforward to apply. Most such methods are based on observation, such as recording data towards a certain phenomenon. Afterwards, data analyzing algorithms are applied for pattern recognition. The target models are designed according to this pattern, which ignores details to some extent. On the other hand, a micro method focuses on the basic rules for each agent. With a careful design, each agent is equipped with certain actions and capabilities, such as interactions with others or with environments. The advantages of micro methods or agent-based modelling lie in its flexibility. To sum up, macro methods are basically based on observation and data, thus unseen situations cannot be learned. Whereas micro methods can create a vast array of situations—including unrealistic ones—to improve the robustness.

The key challenge for agent-based modelling is the design of rules. In natural environments, the habits of creatures are relatively stable, and can be described by observation and analysis. This description, however, is not identical to the true patterns. Further experiments are required in order to discover intrinsic correlations and causations. Rules for agents in artificial systems are also difficult. However, once they are settled approximately optimally, they will contain flexible capability to demonstrate and verify a variety of scenarios.

Fortunately, one reasonable clue for designing rules is that people respond to incentives. This is common to any natural system. People or other creatures tend to the choice for less anxiety. In other words, agents face decision making problems in order to maximize their payoffs. This target motivates us to tend to decision theory and relevant methods, such as game theory and reinforcement learning.

### 3.2. Game Theory and Reinforcement Learning

Applying game theory to solve a resource allocation problem is commonly studied. The key motivation of such research is the existence of the conflict between players or agents. Game theory provides a mathematical solution for a variety of game problems. It extracts models from the real world, considering main factors and ignoring details. The outcomes of any combination of strategies are determined statistically, which may involve the participation and knowledge of humans. By solving different models, game theory solutions are capable of addressing a range of problems in our daily lives, explaining its popularity. Game theory focuses on problems of decision making. It provides a solution for players to follow in order to maximize their payoffs. One major assumption in game theory is that people are rational. This is reasonable, because rational people respond to incentives. In other words, they try to maximize their outcomes. Thus, each player tries to choose the optimal strategy to obtain maximized payoffs. Mathematically, each player *i* tries to find a strategy that satisfies

(5) $$a_{i}^{*}=\max_{a_{i}}U_{i}\left(a_{i},a_{j}\right)$$

where $a_{i}$ is the strategy or action of player *i*, and $U_{i}$ is the payoffs for player *i*. Equation (5) indicates that the best choice for player *i* is the one maximizes $U_{i}$, given the strategy of opponent *j*.

On the other hand, the second fundamental assumption in game theory is that knowledge is common for all players. This assumption seems too strong and impractical in the real world. For instance, two competitive firms may pay great attention to protecting their secrets. Furthermore, even if the knowledge is accessible, determining the values of each outcomes is also costly. This is a critical point, since the values may define the games (according to game theory), which further affects the strategies. Therefore, game theory requires an expensive participation of humans, which may not be directly applicable for practical scenarios.

Fortunately, reinforcement learning—being a branch of machine learning—requires no transparent knowledge. The idea of reinforcement learning originates from psychology and trial-and-error methods. Agents can learn and improve a behavior by interacting with environments and other agents. Reinforcement learning shares natural similarities with game theory, both of which target the problems of decision making. The major advantages of reinforcement learning over game theory is its flexibility and robustness. Specifically, reinforcement learning methods can directly learn from the real event, without extracting models, which decreases the gap between real events and games. Meanwhile, no participation of humans is involved in order to determine the outcomes of certain combinations of strategies. Agents with reinforcement learning methods will learn the outcome if they reach there. Furthermore, games may vary according to different outcomes, leading to different solutions. Thus, a particular game and the following solutions have to be designed towards a certain event. Nevertheless, reinforcement learning methods may be insensitive to particular payoffs. Thus, a general algorithm is able to cover a variety of similar games. Therefore, reinforcement learning methods are more practical and suitable for our market model.

Reinforcement learning considers a Markov decision process (MDP). At each state $s_{t}$ at time *t*, an agent has to choose an action $a_{t}$ according to some policy $a_{t}=\pi \left(s_{t}\right)$ so that her rewards $r_{t}\left(s_{t},a_{t}\right)$ are maximized. One main challenge is that high immediate rewards have no guarantee of optimal global rewards. For instance, a good move in chess cannot ensure the final outcome. Therefore, agents have to take the ability of taking future rewards into consideration. One direct strategy is to discount future rewards so that they are comparable with immediate ones. Thus, the target of reinforcement learning is

(6) $$R=\max E[\sum_{t=0}^{\infty }\gamma^{t}r_{t}\left(s_{t},a_{t}\right)]$$

where *γ* is the discount factor. As a branch of machine learning, reinforcement learning aims to build intelligent thinking machines to replace the work of humans to some degree. Therefore, we only need to equip agents with reinforcement learning methods and to introduce them as a game. After the training process, they will learn a pure or a mixed strategy against their opponents and the environment to obtain the optimal rewards.

It seems that the most popular model-free reinforcement learning method is Q-learning. It is capable of both immediate reward and future reward situations. The general updated rule of Q-learning is

(7) $$\Delta Q\left(s_{t},a_{t}\right)=\alpha \left[r_{t+1}+\gamma max_{a}Q\left(s_{t+1},a_{t+1}\right)-Q\left(s_{t},a_{t}\right)\right]$$

where the current state and action are denoted by $s_{t}$ and $a_{t}$, whereas $s_{t+1}$ and $a_{t+1}$ denote those in the next time period t+1. *γ* is the discount factor, as mentioned previously. *α* is the learning rate and *r* is the reward after action $a_{t}$ is taken. Equation (7) suggests that agents back-propagate the future outcomes, and choose based on the prediction.

Another efficient reinforcement learning method is called learning automata (LA), taking the standard form as

(8) $$\begin{array}{cc} p_{i}\left(t+1\right)=p_{i}\left(t\right)+\lambda_{1}b\left(t\right)\left(1-p_{i}\left(t\right)\right)-\lambda_{2}\left(1-b\left(t\right)\right)p_{i}\left(t\right) & \\ \mathrm{if}\;a\left(t\right)=a_{i} & \end{array}$$

(9) $$\begin{array}{cc} p_{j}\left(t+1\right)=p_{j}\left(t\right)-\lambda_{1}b\left(t\right)p_{j}\left(s\right)+\lambda_{2}\left(1-b\left(t\right)\right)\left(\frac{1}{K-1}-p_{j}\left(t\right)\right) & \\ \mathrm{if}\;a_{j}\neq a_{i} \end{array}$$

where b(t) is the feedback received at time *t*, which is similar to the function of rewards. LA is a model of probability, which means actions are taken with respect to *p*. Different LA methods, such as Linear Reward-Inaction ($L_{R-I}$) or Linear Reward-*ϵ*Penalty ($L_{R-\epsilon P}$) methods are distinguished by the values of $\lambda_{1}$ and $\lambda_{2}$.

One minor problem of applying Q-learning methods to estimate users’ patterns is that Q values are not direct probability. Thus, we apply softmax to converge typical Q-learning into probabilities, as

(10) $$P\left(a\right)=\frac{e^{Q(s,a)/\tau }}{\sum_{i}e^{Q(s,i)/\tau }}$$

where *τ* controls the sharpness of the obtained distribution. Thus, we can apply both Q-learning and LA methods to estimate users’ patterns, which is an expression of probability.

Another advantage of reinforcement learning which is also a central concern is the balance of exploration and exploitation. Agents should be able to stick to the best option, since it maximized their rewards. Simultaneously, they should be able to discover potential higher rewards. This is extremely important for a dynamic environment, such as our real world. It equips reinforcement learning adaptability, so that its robustness is improved. Q-learning methods introduce a ϵ−greedy method or softmax to deal with the tradeoff, whereas LA does not suffer from this problem, since it naturally applies probability to guide policies.

Meanwhile, we can apply reinforcement learning methods to study resource allocation, as the role of game theory. Since we regard the ESNs as a multi-agent environment and we plan to apply an ABM method to study them, we are motivated to equip each single agent with reinforcement learning methods. This is natural and reasonable, since in any multi-agent environment, each agent intends to maximize their payoffs with the lowest cost. Reinforcement learning methods provide them the opportunity of such purpose. This means that if rules are designed according to reinforcement learning methods, resources can be organized according to the choices of each individual, which is similar to the outcomes of a free market. Therefore, we expect to apply price scheme to guide resource allocation, as in economics.

Even though game theory ignores some details when it abstracts models from actual situations, it is still effective and useful to guide resource allocation. Meanwhile, even though a solid proof that reinforcement learning methods can handle game theoretic problems may not exist, they are still capable of solving games. Thus, reinforcement learning methods can be a valid by solution from game theory. Therefore, we design specific games for scenarios such as competition or cooperation to demonstrate the scenarios among agents in ESNs, and to prove the efficiency of applying reinforcement learning methods to allocate resources.

### 3.3. Applying Reinforcement Learning to Estimate Users’ Patterns

Since users’ patterns are unknown (even to users themselves), supervised learning methods, such as support vector machine (SVM), are inapplicable, since the loss function cannot be calculated. This is also because of the inability to label training data. Meanwhile, one interesting advantage of reinforcement learning is its ability to handle uncertain environments. This motivates us to apply reinforcement learning methods to estimate users’ patterns. It is natural for humans and other creatures to discover the mysterious world by interaction. Thus, we borrow a similar idea to describe users’ patterns by providing users with massive data and observing the interactions. For internet applications, this can be achieved by recording users’ internet behaviors, such as searching and using web pages and apps.

Specifically, we initialize a set of probability, and we introduce each agent with data within categories of *N*. Each agent can choose whether to receive, based on his own preferences. If he receives, he obtains a reward of r=1, otherwise r=0. Thus, our probability model is updated according to reinforcement learning methods, and the positive stimulation will increase the value of a certain category and restrain that of the others. We compare the differences between our model with true values of users’ patterns, as estimation error. Notice that the true values are only applied to validate our outcome, instead of being applied to guide our algorithms, because they are actually inaccessible. The dilemma of exploration and exploitation is automatically handled, since the policies of actions are designed according to a probability. This means that even though agents tend to choose the action with highest probability, they still have opportunities to explore.

If the data set is large enough, users’ patterns—which are typically stable over a period of time—can be estimated. Meanwhile, due to the fact that reinforcement learning methods are mostly online, they can handle dynamic situations, which means that even if a huge alteration occurs and patterns are changed, the new patterns can be re-estimated without adaption of the algorithms.

### 3.4. Applying Reinforcement Learning in a Market Model

The market is a natural multi-agent environment, and the intuitive rewards for agents comes from the maximization of their payoffs—if they can manage to make the appropriate choice. In other words, people will purchase the merchant if his reward $R_{m,n}$ is positive, making a successful trade. From the perspective of the data provider, a higher quantity of successful trade *Q* indicates higher profits; namely, profit=Q×P(N),Q≤N. We apply a reinforcement learning method to adjust prices (since it cannot be directly calculated) and compare it to theoretical results. Specifically, data providers choose *Q* within a valid range and obtain *P*, and learn the optimal $Q^{*}$ and $P^{*}$ by interacting with buyers.

Assume that one provider is considered and the strategy she has is the quantity of data. In other words, she could choose any quantity to produce, and receive a response according to her action. Thus, she has to choose wisely in order to maximize her profit. We apply reinforcement learning methods to handle this problem, without considering the details of economic theories. Simulation results will be presented in Section 4.

## 4. Results and Discussion

**Figure 5:**
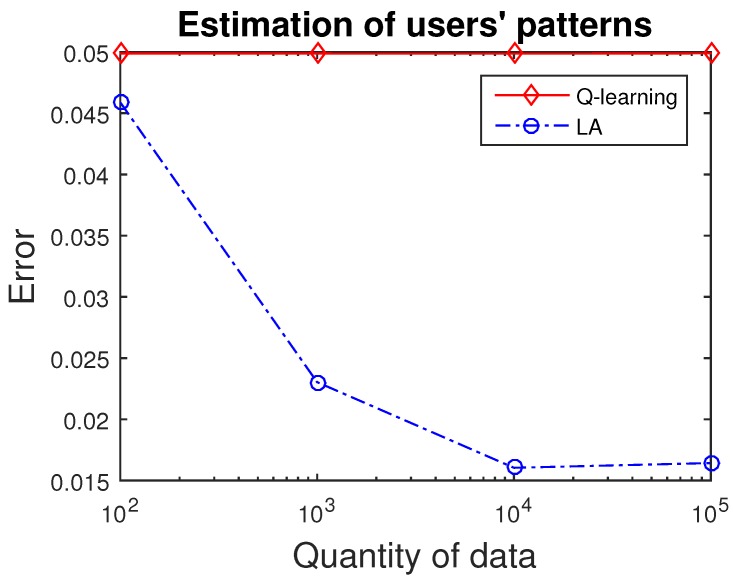
Applying reinforcement learning methods to estimate users’ patterns. The learning automata (LA) method is superior to the Q-learning method, and converges when the quantity of data increases to $10^{4}$.

This section presents our simulation results with discussions attached. All of the experiments were performed on a Windows 2008 R2 Server Enterprise X64 SP1 operating system (Intel Xeon E5-2660 v3 @ 2.60 GHz X2, Hynix DDR4 2133 MHz 256 GB RAM, Kulim, Malaysia) with MATLAB R2014b (from a Dell PowerEdge T630 Main Server Chassis, Xiamen, China). The experiments estimating users’ patterns (Figure 5) took 792.7916 s (about 13 min), since a large number of data were applied. The remaining experiments took only a few seconds.

First of all, we present the result of estimating users’ patterns based on reinforcement learning methods, as in Figure 5. Data are generated randomly over the *N* categories. Users’ patterns indicate the probability of receiving the data. In other words, one tends to possess the data corresponding to the class with the highest probability. Therefore, users’ patterns can be estimated by interacting with data. Intuitively, more data leads to higher accuracy. From Figure 5, it is obvious that both Q-learning and LA methods can achieve an accurate estimation of users’ patterns. With an increase in the amount of data, LA could reduce the accuracy further, whereas Q-learning converged to a relatively high error.

**Figure 6:**
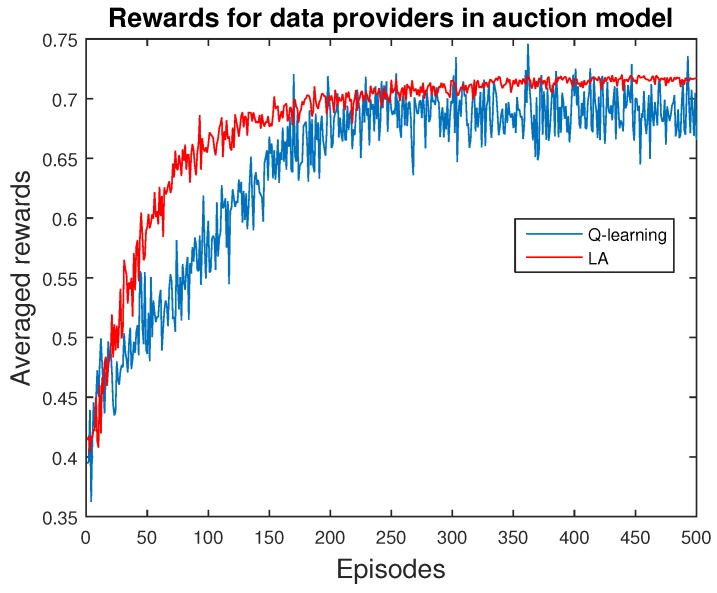
Rewards of data providers in the auction model. It is obviously that agents in the LA method obtain more rewards, indicating that their choices are superior. These learning curves can also demonstrate the efficiency of learning.

**Figure 7:**
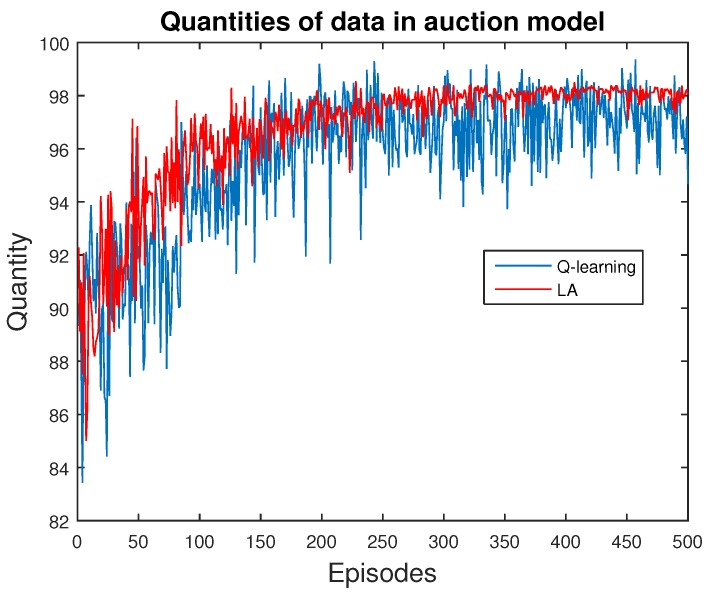
Quantity of data in this market. Notice that the curves are similar to those in Figure 6, due to the method of assigning rewards for each agent.

**Figure 8:**
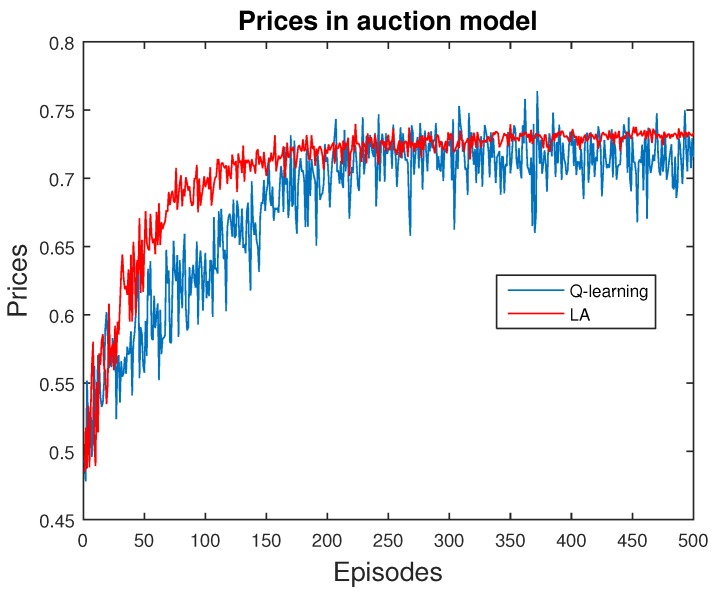
Price in this market. The results are similar to mathematical values.

Next, we present the validation of ABM methods for price scheme. Specifically, we explore the behavior of a single data provider, who chooses the quantity of data and receives the corresponding rewards from the users in this market. We apply both Q-learning and LA methods for comparison, as shown in Figure 6, Figure 7 and Figure 8.

Rewards are a typical factor being examined in the reinforcement learning method, since the key target of an agent is to maximize her rewards. The growing trend of these learning curves indicates the efficiency of the learning algorithms, as in Figure 6. Q-learning is sensitive, leading to a convergence with a high deviation, whereas LA methods can reach a tight and superior outcome with fast convergence, which is superior to the Q-learning method.

We also present the trend of quantity of data supplied, as in Figure 7. When the provider tries to maximize her profit, the quantity of data is also improved. This is natural, because increasing the quantity of selling is always related to improving the profits, until her marginal cost exceeds marginal profit. The final quantity after full convergence is identical to the number of buyers remaining in the market. Thus, resources are allocated within those with the highest preference.

Figure 8 tracks the prices in this market. Theoretically, the optimal price is $P^{*}=W_{D,n}$, as mentioned previously. Thus, we calculate the converged prices from both Q-learning and LA methods as $P_{Q-learning}=0.7058$ and $P_{LA}=0.7242$, whereas the mathematical result is $W_{D,n}=0.7952$. These results indicate the efficiency and accuracy of reinforcement learning methods.

**Figure 9:**
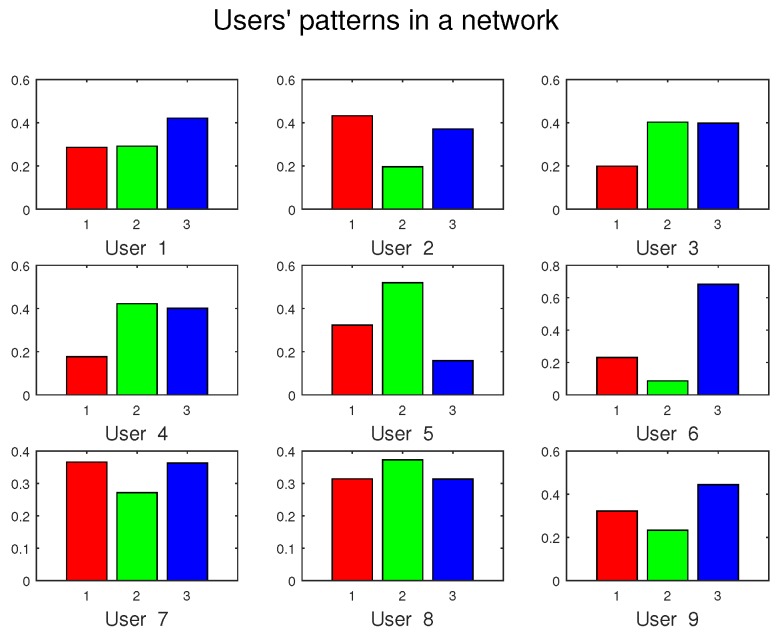
A demonstration of network behaviors. Only nine users with their patterns over three categories of data (distinguished by colors) are presented.

Finally, we present a demonstration of network behaviors. We revisit the model in Figure 1 and expand it in detail, presenting the patterns for each user. Only nine users and three categories of data are taken into consideration, as a simplified instance. Their patterns are generated randomly, as in Figure 9.

Meanwhile, the exact values of their patterns are collected in Table 1. Because columns are preferences over a certain category and the patterns are defined as a set of probability, the sum of each column equals to 1.

Next, the price scheme is introduced. The price of each category of data is initialized as 1. This is reasonable, since a novel object usually has a relatively high price. The price may drop gradually, which takes a pace of 0.01 in our demonstration. People may purchase goods if the price is lower that their willingness-to-pay, which is decided by their patterns. We assume that the number of data exceed the number of buyers; thus, the price may continue to drop so that a large number of people are affordable, whose transaction prices are presented in Table 2.

Due to the differences of their preferences towards a specific category of data, all users are not purchasing simultaneously. When the data are rare and the price is high, only fanatics may buy, increasing their priority for this particular class. Therefore, data are flowed according to their willingness-to-pay and their patterns. Table 3 presents the data flow in our simple demonstration. Thus, the directions of data depend on the patterns of users, and identical networks provide different outcomes according to different stimulations, without further involvement of artificial designs.

Based on previous demonstrations, it is obvious that users’ patterns are the key elements in this model, deciding the different roles of each individual. The model of users’ patterns, however, is still native, which is only an attempt to reveal the general patterns. More sophisticated scenarios may require complicated models or specific models. The competitive relationship among agents in the same layer is widely studied, since the conflict is an interesting topic in decision theory. Both game theory and reinforcement learning have provided a number of research attempts.

Future work will focus on enriching the models for users’ patterns and market models, since our real world is much more complicated than models. Other sophisticated market models (instead of auction models) may also be considered. Maybe the most difficult factor is the rules for agents in ABM. It is true that people respond well to incentives. The real world, however, contains a number of incentives, which may be trustworthy or temptation. Therefore, a sophisticated decision mechanism should be carefully treated, especially when specific scenarios are taken into consideration.

## 5. Conclusions

In this paper, we have presented a market model for resource allocation in emerging sensor networks, which integrates with the environments of the Internet of Things (IoT). By considering the environments as a multi-agent situation, since a number of devices and services are connected, we apply agent-based modelling to study resource allocation problems. After defining the rules of agents with the assistance of reinforcement learning methods, we have managed to define users’ patterns, based on their interaction with others and the environment. Further, we have divided the whole markets into subsets, according to the classes of data. Then, an auction model is introduced to handle the specific markets. Resources are allocated towards high preferences. Finally, the flexibility of applying our methods to guide dynamic topology management is proved. Simulation results demonstrate the efficiency of our methods in these problems.

## Figures and Tables

**Table 1:** Patterns of users in a simple demonstration. The values correspond to the figures in Figure 9.

	User 1	User 2	User 3	User 4	User 5	User 6	User 7	User 8	User 9
Red	0.2866	0.4321	0.1990	0.1766	0.3227	0.2310	0.3659	0.3138	0.3223
Green	0.2920	0.1965	0.4026	0.4224	0.5192	0.0866	0.2718	0.3729	0.2335
Blue	0.4214	0.3714	0.3983	0.4010	0.1580	0.6825	0.3624	0.3133	0.4442

**Table 2:** Transactions price for each user toward the three categories of data.

	User 1	User 2	User 3	User 4	User 5	User 6	User 7	User 8	User 9
Red	0.2800	0.4300	0.1900	0.1700	0.3200	0.2300	0.3600	0.3100	0.3200
Green	0.2900	0.1900	0.4000	0.4200	0.5100	0.0800	0.2700	0.3700	0.2300
Blue	0.4200	0.3700	0.3900	0.4000	0.1500	0.6800	0.3600	0.3100	0.4400

**Table 3:** Data flow in this demonstration. Orders are naturally established based on their patterns.

	1st	2nd	3rd	4th	5th	6th	7th	8th	9th
Red	User 2	User 7	User 5	User 9	User 8	User 1	User 6	User 3	User 4
Green	User 5	User 4	User 3	User 8	User 1	User 7	User 9	User 2	User 6
Blue	User 6	User 9	User 1	User 4	User 3	User 2	User 7	User 8	User 5
